# Muscle Progenitors Derived from Extraocular Muscles Express Higher Levels of Neurotrophins and their Receptors than other Cranial and Limb Muscles

**DOI:** 10.3390/cells9030747

**Published:** 2020-03-18

**Authors:** Génova Carrero-Rojas, Beatriz Benítez-Temiño, Angel M. Pastor, Mª América Davis López de Carrizosa

**Affiliations:** Department of Physiology, Universidad de Sevilla, 41012 Seville, Spain; carrero92@gmail.com (G.C.-R.); bbtmino@us.es (B.B.-T.); ampastor@us.es (A.M.P.)

**Keywords:** extraocular muscles, neurotrophins, BDNF, NGF, NT-3, p75^NTR^, Trk, skeletal muscle, satellite cells, myogenic progenitors, myogenesis, regeneration

## Abstract

Extraocular muscles (EOMs) show resistance to muscle dystrophies and sarcopenia. It has been recently demonstrated that they are endowed with different types of myogenic cells, all of which present an outstanding regenerative potential. Neurotrophins are important modulators of myogenic regeneration and act promoting myoblast proliferation, enhancing myogenic fusion rates and protecting myotubes from inflammatory stimuli. Here, we adapted the pre-plate cell isolation technique to obtain myogenic progenitors from the rat EOMs, and quantified their in vitro expression of neurotrophins and their receptors by RT–qPCR and immunohistochemistry, respectively. The results were compared with the expression on progenitors isolated from buccinator, tongue and limb muscles. Our quantitative analysis of brain-derived neurotrophic factor (BDNF), nerve growth factor (NGF) and neurotrophin-3 (NT-3) transcripts showed, for the first time, that EOMs-derived cells express more of these factors and that they expressed TrkA, but not TrkB and TrkC receptors. On the contrary, the immunofluorescence analysis demonstrated high expression of p75^NTR^ on all myogenic progenitors, with the EOMs-derived cells showing higher expression. Taken together, these results suggest that the intrinsic trophic differences between EOMs-derived myogenic progenitors and their counterparts from other muscles could explain why those cells show higher proliferative and fusion rates, as well as better regenerative properties.

## 1. Introduction

Extraocular muscles (EOMs) are a group of craniofacial skeletal muscles that are responsible for eye movements. Highly specialized, they are fundamentally distinct from other muscles and thus have been classified as a separate muscle allotype [1]. EOMs differ from other cranial, limb and body muscles developmentally, anatomically, structurally, biochemically and physiologically [2,3,4,5]. They even respond differently to aging [6] and to certain disorders affecting specifically the skeletal muscle, remaining anatomically and functionally intact even at the late stages of Duchenne muscular dystrophy (DMD) in humans [7] and in animal models of this disorder [8].

Some of the biochemical and physiological differences of the EOMs have been proposed as plausible causes behind their sparing in some dystrophies and sarcopenia [7,9,10]. However, in recent years, the discovery of the unique properties of EOM myogenic progenitors and their proliferative and regenerative features [11,12,13] point them as fundamental factors contributing to the normal EOM structure and function in DMD and other muscle disorders.

Adult skeletal muscles present a high regenerative potential due to the presence of a population of myogenic progenitors that express the transcription factor Pax7 [14] and reside in the muscle niche as satellite cells (SCs) [15] for their location inside the basal lamina of muscle myofibers. Although it was first thought that these stem cells were in a quiescent state and only became activated by signals related to muscle damage [16,17], recent studies have demonstrated the continuous fusion of SCs to healthy myofibers in different adult mice muscles [18,19,20]. Noticeably, Keefe and collaborators also found that EOM SC density is fivefold higher than in most limb muscles and that these cells continuously contribute to the formation of new EOM myofibers even in the aged mice [18]. In vitro experiments reveal EOM SCs possess higher proliferative and renewal features not only in culture assays but also when engrafted into host *mdx* mice [13]. Taken together, their higher density and proliferative rates make them ideal candidates in cell-based therapies to withstand disorders affecting skeletal muscles. 

Neurotrophic factors are a group of well described proteins produced and secreted by numerous cells. Essential for the correct development of the nervous and motor systems [21,22,23], these signaling molecules also exert important roles regulating and maintaining several functional and morphological traits of different adult populations in these systems [24,25,26,27]. 

An important subgroup of neurotrophic factors is the neurotrophin family which includes nerve growth factor (NGF), brain-derived neurotrophic factor (BDNF), neurotrophin-3 (NT-3) and neurotrophin-4/5 (NT-4). Some of these molecules have been recently implicated in different aspects of the myogenic process [27]. Of special interest is the role of BDNF and NGF in myogenesis and regeneration of the skeletal muscle. Although the profile of expression and the role of BDNF in development and adult skeletal muscle remains controversial [26], some experiments have demonstrated that the expression of BNDF in SCs of adult mice skeletal muscle is essential for proper muscle regeneration after injury [28,29,30]. In humans, BDNF is normally produced by precursor and differentiated muscle cells, and BDNF gene silencing or protein blockade in cultured myoblast also hampers myogenesis [31].

Although controversial data are available, several lines of evidence show that NGF is also involved in muscle regeneration. In fact, direct stimulation to muscle stem cells with this neurotrophin significantly reduced their in vitro differentiation ability and enhanced the cells’ engraftment efficiency when transplanted in dystrophic muscle of the *mdx* mice [32]. The molecular mechanisms guiding myoblast fusion to damaged myofibers and muscle repair in vivo depend on the NGF- p75^NTR^ signaling pathway [33], which inhibits the GTPase RhoA [34,35]. In addition, a transgenic mice model expressing a neutralizing antibody against NGF in the adult, resulted in dystrophy of skeletal muscle [36,37].

Finally, only a few studies have focused their attention on NT-3 role in skeletal muscle physiology [38,39]. Recently, Yalvac et al. [40] demonstrated that NT-3, acting through direct activation of the mTOR-TrkC related pathway, increases muscle fiber diameter in the neurogenic muscle from the Trembler-J mice. 

In this study, we compared the in vitro expression of neurotrophins and their receptors in myogenic progenitors derived from different cranial and limb muscles. We aimed to investigate if there are intrinsic trophic differences between these progenitors that could explain why the EOMs SCs show higher proliferative and fusion rates and better engraftment efficiencies, features that seem to be in part responsible for the EOM resistance to aging and certain diseases. 

## 2. Materials and Methods

All experiments have been conducted in adult Wistar rats according to Spanish law (R.D. 53/2013, BOE 34/11370-421) and international guidelines of the European Union (2010/63/EU) for the use and care of laboratory animals. Animal care and experimental procedures were approved by the ethics committee of the Universidad de Sevilla.

In this article, the in vitro expression of different neurotrophins and their receptors were compared in myogenic progenitors derived from the EOMs, the intrinsic muscles of the tongue, the buccinator and the extensor digitorum longus (EDL) by means of RT–qPCR and immunohistochemistry, respectively.

### 2.1. Tissue Harvesting

For each culture, cells were obtained from the EDLs, intrinsic muscles of the posterior part of the tongue, buccinators and the EO recti muscles of 2 female rats, typically 2 or 3 months old. To obtain the EO recti muscles and EDLs, we followed the procedures described by Stuelsatz et al. [41] and Keire et al. [42], respectively. The tongue was excised close to the epiglottis using sharp scissors and placed on a Petri dish with sylgard^®^ 184 (Sigma Aldrich, St. Louis, MO, USA) for further dissection of the intrinsic musculature. Connective tissue, glands, fat, epithelium and mucosa were discarded and muscle bundles of the posterior part were dissected. To access the buccinators, the skin surrounding the snout was removed, masseter, levator, caninus and zygomaticus muscles were cast aside and the insertions of the buccinator muscle were cut. After tissue harvesting, every muscle was thoroughly cleaned to remove connective tissue, fat and blood vessels and EDLs and tongue muscles were minced into small pieces using surgical forceps and scissors.

### 2.2. Isolation and Culture of Myogenic Progenitors

To isolate the myogenic cells, we applied a slightly modified protocol of that described by Rando and Blau in 1994 [43]. Briefly, after harvesting, every muscle was collected in 500 µL of recollection medium (high glucose DMEM, Sigma Aldrich, St. Louis, Mo, USA) containing 1% glutamax, 1% penicillin and streptomycin and 1% of amphotericin B (all from GIBCO BRL, Gaithersburg, MD, USA) and weighed to subsequently quantify the number of cells obtained per gram of tissue.

Whole (buccinator and EO) or minced (EDL and tongue) muscles were digested with Pronase E (1.4 mg/mL) in dissociation medium (high glucose DMEM:Ham’s F12 [1:1]), 15 mM of HEPES (all from Sigma Aldrich, St. Louis, MO, USA), 1% of penicillin and streptomycin and 1% of amphotericin for 1 h at 37 °C. Mechanical trituration was applied every 15 min to homogenize the tissue-enzyme mixture and to release cells from the muscle fibers. After digestion, cell suspension was filtered through a 40 µm cell strainer and cells centrifuged at 1000× *g* for 10 min.

To lyse erythrocytes, cell pellets were suspended in 1ml of Red Blood Cells lysis buffer (Roche Molecular Systems, Pleasanton, CA USA) in ice for 2.5 min. The same volume of growth medium (high glucose DMEM with 1% of glutamax, 20% of fetal bovine serum, 10% of horse serum and 1% of chicken embryo extract, 1% penicillin and streptomycin and 1% amphotericin B), was added and cells were pelleted at 500× *g* for 5 min and then suspended in 500 µL of growing medium. All of the sera were obtained from Thermo Fisher Scientific, USA. The final pellet was first plated on uncoated plates, named PP1 (pre-plate 1), with growing medium and, after 2 h, floating cells were transferred to rat tail type I collagen-coated plates (50 µg/mL, GIBCO BRL, Gaithersburg, MD, USA), named PP2. All cultures were maintained in a 5% CO_2_ humidified tissue incubator at 37 °C. After 72 h, cells were rinsed with DMEM to eliminate debris and from that moment on, the growing medium was replaced every 48–72 h. 

### 2.3. Density of Mononuclear Cells 

The density of mononuclear cells on each type of muscle (number of cells/g of muscle dissected) was obtained by quantifying the number of fresh cells isolated from each type of muscle before they were first plated and comparing the result with the weight of harvested muscles, after removing connective tissue, fat and blood vessels, this is, right before the digestion. Quantification of cells was performed for each type of muscle in a Neubauer chamber staining 10 µL of cells with 10 µL of Trypan Blue (GIBCO BRL, Gaithersburg, MD, USA). 

In order to compare the number of cells retained all along our isolation and pre-plating protocol, we compared the number of freshly obtained cells before first plated, with the number of cells obtained 3 to 4 days after plating on PP2 for each type of muscle.

### 2.4. RNA Extraction, cDNA Synthesis and Real-Time PCR for the Detection of Neurotrophins

The expression of BDNF, NGF and NT-3 in myogenic progenitors derived from EO, tongue, buccinator and EDL muscles were analyzed by qPCR. For this purpose, adherent cells from PP2 were trypsinized after one week in culture with 0.05% trypsin (Sigma Aldrich, St. Louis, Mo, USA) in PBS, counted and total mRNA from the cell suspension was extracted following the protocol for the RNeasy Plus Micro Kit (Qiagen, Venlo, Netherlands). The reverse transcription polymerase chain reaction to obtain cDNA was carried out with the QuantiScript Reverse Transcription Kit (Qiagen, Venlo, Netherlands) using random hexamers and the oligo (dT) primer provided with the kit, and a T100 Termocycler (BioRad, Berkeley, CA, USA). Concentration and pureness of the samples were determined using the spectrophotometer NanoDrop 2000 (Thermo Scientific, Walthan, MA, USA). All cDNA samples were stored at −20 °C at a final concentration of 100 ng/µL. Finally, qPCR was used to amplify specific cDNA of the above-mentioned trophic factors, obtained from the different myogenic progenitor populations.

Briefly, reaction mixtures (10 µL) were prepared according to the kit guidelines (SensiFAST SYBR; Bioline, London, UK). A 1 µL volume of each sample was incubated with specific primers designed to bind either BDNF, NGF or NT-3 as target genes or actin-b (Act) or phosphoglycerate kinase-1 (PGK-1) as housekeeping genes. All reactions were run in triplicates using LightCycler 480 equipment (Roche Molecular Systems, Pleasanton, CA, USA). The qPCR protocol included a pre-denaturation step (95 °C for 2 min) and 40 reaction cycles including the following steps: denaturation (95 °C for 5 s), annealing (60 °C for 13 s) and extension (72 °C for 7 s). The specificity of the amplificated product was assessed by a melt curve analysis using LightCycler 480 software. Threshold cycles (Ct) were determined by the second derivative of the fluorescence curve. Relative quantification using ∆∆Ct was carried out and data were relativized to EOM results.

Predesigned Act, PGK-1, BDNF and NGF primers were obtained from PrimePCR Assays and ControlsBioRad (Act, qRnoCID0056984; PGK-1, qRnoCED0002588; BDNF qRnoCED0005012; NGF qRnoCID0003911, respectively). NT-3 primer was custom-designed using sequence databases (NCBI, USA) and free software (OligoCalc, Biotools, USA; Fwd: 5’- CCGAACTCGAGTCCACCTTT -3’; Rv: 5’-AATTACCAGAGCACCCTGCC -3’, NCBI Reference Sequence: NM_031073.3).

### 2.5. Immunohistochemistry and Immunofluorescence

When cells reached 70–80% confluence, 3 or 4 days approximately, depending on the muscle and initial quantity of cells plated, they were subcultured with 0.05% trypsin (Sigma Aldrich, St. Louis, Mo, USA) in PBS and plated with growth medium on collagen-coated coverslips at a density of 10 × 10^3^ cells/cm^2^ and incubated at 37 °C. After 24–48h, cells were washed with DMEM, fixed in 4% paraformaldehyde mixed with the same volume of DMEM for 10 min and then rinsed extensively with PBS. For the immunocytochemistry, cells were first permeabilized in PBS with 0.5% triton (PBS-T), then blocked 1 h in blocking solution containing 5% normal donkey serum, 2.5% bovine serum albumin, 0.05% Tween-20 in PBS with 100 mM glycine and finally incubated overnight at 4 °C with the corresponding primary antibodies. All the primary antibodies were diluted in blocking solution without glycine. 

To evaluate the myogenicity of our primary cultures, different markers of the myogenic lineage were used: Pax7 (mouse anti-Pax7; diluted 1:20; Developmental Studies Hybridoma Bank, Iowa City, IA, USA), MyoD (mouse anti-MyoD; diluted 1:200; BD Bioscience, La Chaux-de-Fonds, Switzerland) Myogenin (mouse anti-Myogenin; diluted 1:75; Developmental Studies Hybridoma Bank, Iowa City, IA, USA) and desmin (rabbit anti-desmin; diluted 1:50; Jackson ImmunoResearch, UK). The latter antibody was always used in combination with one of the former nuclear antibodies. 

To study the expression of the neurotrophin receptors on cultured cells, primary antibodies against TrkA, TrkB and TrkC were used at the following concentrations: rabbit anti-TrkB [1:100]; rabbit anti-TrkA [1:50]; rabbit anti-TrkC [1:50], respectively; all from Santa Cruz Biotechnology, USA. The expression of the receptor p75^NTR^ was studied using a rabbit anti-p75^NTR^ [1:200] antibody kindly provided by Dr. Louis Reichardt (University of California, San Francisco, CA, USA). After incubation with the primary antibody, cells were rinsed with PBS-T and incubated with the corresponding secondary antibody in PBS-T for 2 h; FITC or TRITC donkey anti-mouse was used for the myogenic markers and receptors (diluted 1:250 and 1:50, respectively, Jackson ImmunoResearch). In all cases, nuclei were stained with 4′,6-diamidino-2-phenylindole (DAPI, diluted 1:10^4^; Sigma Aldrich, St. Louis, Mo, USA) and the cytoskeleton with Phalloidin-Atto633 (diluted 1:200; Thermo Fisher Scientific, Walthan, MA, USA) in PBS for 15 min. Finally, coverslips were mounted on slides with DAKO fluorescence mounting medium (Agilent, Santa Clara, CA, USA). For negative controls, an identical procedure was performed with omission of the primary antibody.

### 2.6. Confocal Microscopy and Image Analysis

Fluorescence images were captured with a confocal laser-scanning microscope Zeiss LSM 7 DUO (Zeiss, Oberkochen, Germany) and ZEN lite software. UV 405 nm, argon 488 nm, DPSS 561 nm and HeNe 633 nm lasers were used to excite DAPI, FITC or Cy2 fluorophores with 488-nm argon laser, TRITC with 534 nm laser and atto633 wavelengths, respectively.

For the analysis of the percentage of cells expressing the different combinations of the myogenic markers (Pax7-desmin, MyoD-desmin, Myogenin-desmin), on each coverslip (2 per type of muscle and culture, 2 independent cultures), 8-bit images of 6 independent random fields visualized in the DAPI channel with the 20× objective from each muscle were captured. Images having less than 10 cells were discarded. Using ImageJ software, on each image, the number of Pax7^+^, MyoD^+^ or Myogenin^+^ nuclei, desmin^+^ cytoplasms and the total number of cells (DAPI^+^) was counted and the percentage of cells expressing the myogenic markers was determined.

For the confocal analysis of neurotrophin receptors on cultured cells, to capture the images, all the parameters of the lasers were equally set. The expression of the Trk or p75^NTR^ receptors was analyzed from 6 randomly chosen confocal images as described above for the myogenic markers. The average optical density (OD) of immunofluorescent cells was calculated using ImageJ software. For p75^NTR^ and TrkA receptors, the signal intensity of the FITC channel was measured on 40× images by outlining the cytoplasm of cells, nucleus excluded, for the former, and by outlining the DAPI^+^ area for the latter. In both cases, for background correction, five OD readings were taken per image in areas devoid of cells. Then, the OD value of every cell was divided by the mean background level determined for the same image. Only cells having at least two times the background level were included in the analysis. Data were presented as the mean percentage of the times above the background of the OD of the receptor-positive cells from each muscle and values were relativized to the same parameter on EOM-derived cells. 

### 2.7. Statistics

Data were statistically compared using the one-way-ANOVA test followed by post-hoc multiple comparisons (Holm–Sidak method) at a significant level of *P* < 0.05. All values are expressed as the mean ± standard error of the mean (SEM). For populations without a normal distribution, a non-parametric test was used (Kruskal–Wallis followed by Dunn’s method for post-hoc comparisons). A paired *t*-test at a significant level of *P* < 0.05 was used to compare the number of cells derived from each muscle before plating and after 4 days in culture. Statistical analysis was carried out in Sigma Plot, version 11 (Systat Software, San José, CA, USA).

## 3. Results

### 3.1. Extraocular and Buccinator Muscles Show Higher Mononuclear Cell Densities when Compared with EDL and Tongue Muscles

In order to compare the relative quantity of mononuclear cells obtained from each type of muscle, we weighed the harvested and cleaned muscles before digestion and, after applying the same isolation protocol on every muscle, we quantified the number of freshly isolated cells. Sampled muscle’s weight, obtained from two animals on each independent culture, is shown in the first row of Table 1. We found no differences between the weight of the EDLs and tongue muscles, however, the EOM weighted less than half the value of the former muscles (one-way ANOVA test: *F*(_3,17_) = 122.578, *P* < 0.001, Holm–Sidak method for post-hoc comparison, *P* < 0.05). Remarkably, we found no differences in the number of cells isolated from these three muscles (second row on Table 1, one-way ANOVA test: *F*_(3,17)_ = 5.061 *P* < 0.011, Holm–Sidak method for post-hoc comparison, *P* < 0.05). The weight and number of cells derived from the buccinator muscle were significantly lower than those obtained from the other muscles. The relative number of mononuclear cells on each muscle was obtained dividing the number of fresh cells by the weight in grams of each muscle (Row 4 of Table 1). Our results showed that the buccinator had a higher density of cells per gram of muscle, reaching 2.21 × 10^5^ cells/g muscle. EOMs showed lower densities (1.45 × 10^5^ cells/g muscle) but still significantly higher than EDL and tongue muscles (one-way ANOVA test: *F*_(3,17)_= 10.998, *P* < 0.001, Holm–Sidak method for post-hoc comparison, *P* < 0.05). Finally, we estimated the number of animals that would be needed to obtain 1 × 10^6^ of freshly isolated cells from each type of muscle. Our results indicate that buccinators would be the less efficient muscle to obtain high yields of cells, while EOM, although light in weight, provided considerably more cells (final row in Table 1).

No significant differences were found in the number of cells obtained before first plating and the cells obtained on PP2 in any of the muscles (Rows 2 and 3 of Table 1; *t*-test, t_(15)_ = −0.0615, *P* = 0.952 for EOM, *t*-test, t_(6)_ = −0.209, *P* = 0.842 for buccinator, *t*-test, t_(5)_ = 0.402, *P* = 0.704 for tongue and *t*-test, t_(4)_ = 0.382, *P* = 0.722 for EDL). These results suggest that, for all of the muscles tested, the number of cells lost on PP1 was similar to the number of cells obtained by proliferation in the first three to four days in culture.

### 3.2. Myogenicity of the Primary Cultures and State of Differentiation

**Figure 1:**
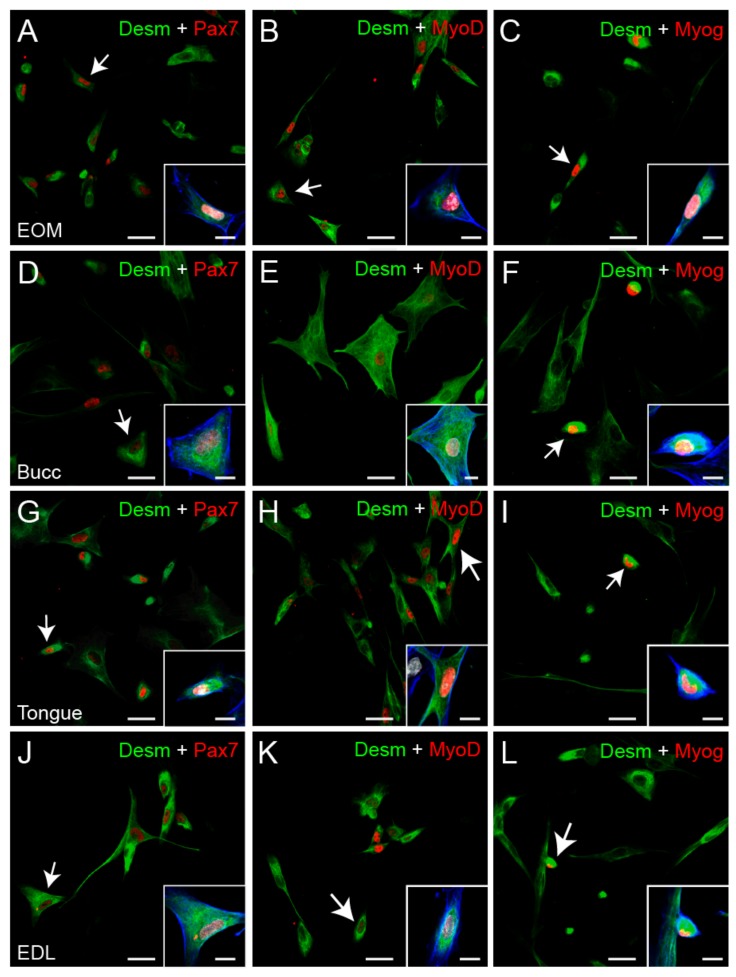
Myogenicity of the cultured cells. Confocal images showing cultured cells obtained from each type of muscle and immunostained for the myogenic markers (Pax7, MyoD and Myogenin (Myog): red, and desmin (Desm): green, as indicated on each image). Insets show a magnified image of the cell indicated by the white arrow on each case. In these images, the cytoplasm is marked with phalloidin (blue) and the nucleus with DAPI (white). Images (**A**–**C**), (**D**–**F**), (**G**–**I**) and (**J**–**L**) correspond to cells obtained from EOM, buccinator (Bucc), tongue and EDL, respectively. Arrows indicate cells immunopositivity for two markers. Scale bars: A to L: 25 μm, inset on each image: 10 μm.

**Figure 2:**
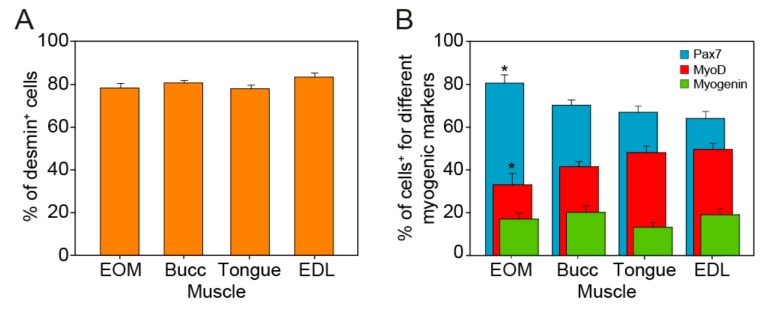
Percentage of myogenic cells obtained from the muscles under study. (**A**) Bar charts comparing the mean and SEM of the percentage of desmin^+^ cells on progenitors derived from each muscle. (**B**) Bar charts comparing the mean and SEM of the percentage of immunostained cells for each marker (Pax7: blue, MyoD: red, Myogenin: green) on progenitors derived from each muscle. Asterisk (*) indicates significant differences with cells derived from tongue and EDL (one-way ANOVA, Holm–Sidak method for post-hoc comparisons, *P* < 0.05).

To further test the validity of our isolation protocol, we evaluated the myogenicity of the cells from each type of muscle by means of double immunocytochemistry against the myogenic transcription factors: Pax7, MyoD, or Myogenin, combined with the cytoplasm myogenic marker desmin. Two independent cultures obtained from each of the four sampled muscles were used for this analysis. Notably, as shown in Figure 1 and Figure 2A, the great majority of cells were immunopositive for desmin, demonstrating that our cultures were enriched with myogenic precursors. 

When the percentage of Pax7^+^ and MyoD^+^ cells were compared, the EOMs showed 80.6% ± 3.8%, *N* = 24 and 33.2% ± 5.3, *N* = 24, respectively, while cultures derived from the tongue and the EDL showed a lower percentage of cells expressing Pax7; 67.0% ± 2.8%, *N* = 24 and 64.4% ± 3.2%, *N* = 25, respectively and a higher percentage of cells expressing MyoD; 48.2% ± 3.1%, *N* = 23 and 49.7% ± 2.8%, *N* = 23, respectively (Figure 2B). No differences were found in the percentage of Pax7^+^ and MyoD^+^ progenitors in cultures derived from the buccinator when compared with progenitors derived from the other muscles (70.5% ± 2.4%, *N* = 24 and 41.6% ± 2.4%, *N* = 24, respectively, one-way ANOVA test, F_(3,93)_ = 5.185, *P* = 0.002; one-way ANOVA test, F_(3,91)_ = 4276, *P* = 0.007, respectively). In all of the cultures, the proportion of differentiating myoblast (Myogenin^+^) varied between 10% and 20% of the cells, and no differences between groups were found (Figure 2B, green bars EOM: 17.1% ± 2.8%, *N* = 24, buccinator: 20.2% ± 3.1%, *N* = 25, tongue: 13.4% ± 2.2%, *N* = 23, EDL: 19.2% ± 3.0%, *N* = 23, one-way ANOVA test, F(_3,91_) = 1.129, *P* = 0.342).

Overall, these results confirm that, relative to their weight, the EOMs are endowed with higher densities of mononuclear cells, 80% of which were myogenic and that the adapted protocol used here is an efficient technique to isolate these cells.

### 3.3. Myogenic Progenitors Derived from the EOMs Express More Neurotrophins

**Figure 3:**
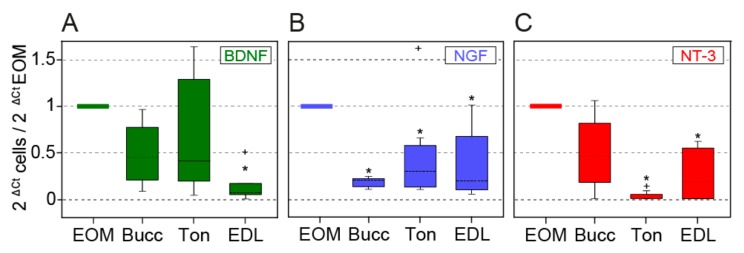
Expression of neurotrophins on cultured cells quantified by RT–qPCR. (**A**) Box-and-whisker plots summarizing RT–qPCR results on expression of brain-derived neurotrophic factor (BDNF) (A), nerve growth factor (NGF) (**B**) and neurotrophin-3 (NT-3) (**C**) on cells derived from EOM, buccinator (Bucc), tongue (Ton) and EDL. The boxes show the 25th, 50th (median) and 75th percentiles, and the whiskers represent the 10th–90th percentile. Plus sign (+) shows data points considered outliers (data outside 10th–90th percentile). Data are normalized with respect to those obtained from EOM cells. Asterisk (*) indicates significant differences with cells derived from EOM.

In order to compare the expression of neurotrophins between the studied myogenic populations, an RT–qPCR analysis was performed and it revealed several differences. Thus, BDNF expression in cells derived from the buccinator and tongue muscles was approximately 50% of the expression detected in EOM cells (Figure 3A, median relative values = 1, 0.46, 0.41 for EOM, buccinator and tongue cells, respectively, *N* = 11, 4, 8), although no statistical differences were found (Kruskal–Wallis followed by Dunn’s post-hoc comparison). EDL cells showed a lower expression when compared to EOM (median = 0.07; *N* = 5; Kruskal–Wallis, *P* = 0.004; Dunn’s post-hoc comparison, *P* < 0.05). In the case of NGF (Figure 3B), EOM cell expression (median relative value of 1) was higher than that observed in buccinator (0.20), tongue (0.16) or EDL cells (0.19); (Kruskal–Wallis, *P* < 0.001; Dunn’s method for post-hoc comparison, *P* < 0.05). The expression of NT-3 (Figure 3C) was also higher in EOM cells (median value of 1) than in tongue and EDL precursor cells (0 and 0.19, respectively; Kruskal–Wallis, *P* < 0.001; Dunn’s method for post-hoc comparison, *P* < 0.05). NT-3 expression in buccinator cells (0.46) did not differ from that observed in EOM cells.

### 3.4. Expression of Trk and p75^NTR^ Receptors Shown by Immunofluorescence

**Figure 4:**
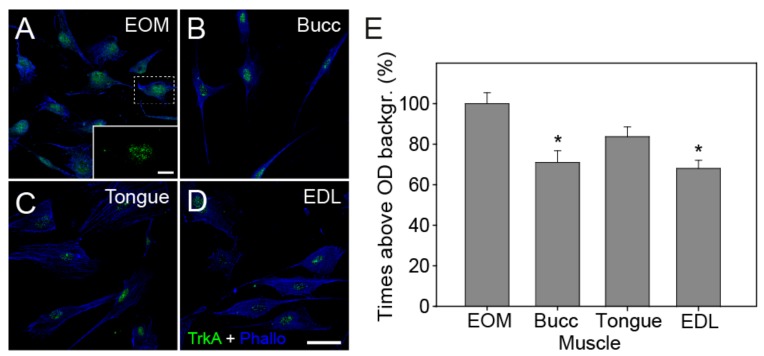
Expression of TrkA and quantification of the optical density (OD). (**A–D**) Confocal images showing the immunostaining for TrkA receptor (green) and the cytoplasm stained with phalloidin (blue). Images A to D correspond to cells obtained from EOM, buccinator (Bucc), tongue and EDL, respectively. Scale bars: 40 μm. (**E**) Bar charts comparing mean ± SEM times above OD background for TrkA immunofluorescence in cells obtained from each muscle, expressed as a percentage with respect to EOM-derived cell values (number of cells analysed: 126, 78, 101 and 79 for EOM, buccinators, tongue and EDL, respectively). The asterisks (*) indicate significant differences on the expression of this receptor in cells derived from buccinator and EDL when compared with EOM.

We studied by means of immunocytochemistry the in vitro expression of its receptors on cultured cells derived from each of the studied muscles. No expression of the TrkB and TrkC receptors was found on any of the myogenic precursors analyzed. Expression of the TrkA receptor was limited to the nuclei of the myogenic progenitors with almost no expression on the cytoplasm of the cells (Figure 4A–D). Analysis of the OD demonstrated that cells derived from the buccinator and EDL muscles showed significantly less expression of the NGF receptor TrkA (71.0% ± 5.8%, *N* = 78 and 68.4% ± 4%, *N* = 79, respectively) when compared with progenitors derived from the EOMs (100% ± 5.4%, *N* = 126; Figure 4E, one-way ANOVA test, F(_3,380_) = 8.203, *P* < 0.001). 

**Figure 5:**
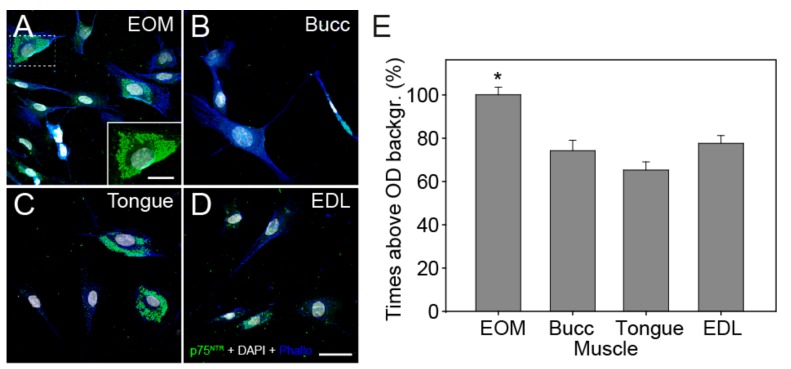
Expression of p75^NTR^ and quantification of the optical density (OD). (**A–D**) Confocal images showing the immunostaining for the p75^NTR^ receptor (green) and the nuclei stained with DAPI (white). Images (A–D) correspond to cells obtained from EOM, buccinator (Bucc), tongue and EDL, respectively. Scale bars: 40 μm. (**E**) Bar charts comparing the mean ± SEM times above OD background for p75^NTR^ immunofluorescence in cells obtained from each muscle, expressed as the percentage with respect to EOM cell values (number of cells analyzed: 242, 90, 110 and 126 for EOM, buccinators, tongue and EDL, respectively). The asterisk (*) indicates significant differences in the expression of this receptor in cells derived from EOM when compared with the other muscles.

Neurotrophins may also exert their actions through the low-affinity receptor p75^NTR^. Analysis of its expression demonstrated that all the myogenic progenitors expressed this receptor, but not with the same intensity (Figure 5A–D). Myogenic progenitors derived from the EOMs showed significantly higher optical densities when compared with the cells derived from the other muscles (Figure 5E, 100% ± 3.5% on EOM, *N* = 242; 74.31% ± 4.8% on buccinator, *N* = 90; 65.38% ± 3.7% on tongue, *N* = 110; 77.69% ± 3.6% on EDL cells, *N* = 126, respectively (one-way ANOVA test, F(_3,564_) = 16.960, *P* < 0.001).

## 4. Discussion

Here, we aimed to investigate if there were intrinsic trophic differences between muscle progenitors derived from the EOMs, the muscles of the tongue, the buccinator and the EDL, that could explain why the EOMs SCs show higher proliferative and fusion rates (reviewed by [44]), features that seem to be responsible for the extraordinary EOMs regenerative properties and their resistance to certain diseases [11,13]. 

We first confirmed that EOMs are endowed with high densities of mononuclear cells. Although the weight of the EOMs processed for each culture to isolate their mononuclear cells was less than half the weight of the EDLs and tongue muscles, there were no differences in the number of freshly isolated cells obtained from these three muscles (Table 1, Rows 1 and 2). It has been described that these freshly isolated mononuclear cells consist of hematopoietic and endothelial cells, mesoangioblast, pericytes, fibroblast and several populations of muscle-derived stem cells, all of which contribute to the muscle regeneration process [45]. Interestingly, the number of living cells obtained did not vary for any of our studied muscles from the moment they were isolated to their first passage from PP2 (Table 1, Rows 2 and 3). This could suggest that the number of cells lost on pp1, essentially fibroblast, pericytes and other non-adherent cells, was substituted by cells obtained by proliferation during those first three to four days in culture. certainly, the cellular composition of the cultures was different, since the pre-plate technique ensures the elimination of most of the non-myogenic cells in the passage from pp1 to further plates [46], but it would be of interest to further study if there are differences in the populations isolated from each type of muscle. In this regard, we analyzed the percentage of cells expressing the paired box transcription factor, pax7, and the mrfs desmin, myod and myogenin. Each of these mrfs is expressed and directs distinct stages of the scs during skeletal myogenesis [47]. Thus, pax7 is expressed in quiescent and activated scs and, to a lesser extent, in proliferating myoblast. The later cells are characterized by the upregulation of the transcription factor myod and later, when they are committed to myogenic differentiation and start to fuse to existing fibers or other myocytes, by the expression of myogenin [48]. Interestingly, approximately 80% of the cells derived from each type of muscle were desmin^+^ (Figure 2A), percentages similar to those obtained by others using similar adhesion-based techniques [49,50], demonstrating the validity of our adapted protocol to obtain myogenic progenitors from these rat muscles. However, we found dissimilarities in the differentiation state of the cells obtained from the EOMs as compared with the other muscles. While the former muscles yielded 80% of Pax7^+^ cells, 33% of MyoD^+^ cells and only 17% of Myogenin^+^ cells, the EDL, for example, yielded 64%, 49% and 19% of cells expressing these markers (Figure 2B). Higher values of Pax7^+^ cells and lower levels of MyoD and Myogenin^+^ cells may suggest that the stemness of SCs derived from EOMs is higher when compared with their counterparts from other muscles. Moreover, it has been demonstrated that the EOMs have a unique second large population of stem cells contributing to their regeneration [51]. These myogenic cells expressing the homeobox transcription factor Pitx2, are negative for the SCs Pax7 canonical marker and they seem to be important contributors to the regenerative capacity of the EOMs [12]. It cannot be ruled out that at least part of the remaining 20% of Pax7^−^ cells obtained from the EOMs could be part of this second myogenic population.

In addition, since it is demonstrated that EOM-derived cells fulfill all the criteria to be excellent candidates for cell-based therapy in skeletal muscle disease and sarcopenia [11,13], we estimated the number of animals needed to obtain approximately 1 × 10^6^ of freshly isolated cells from each type of muscle (final row on Table 1). Thus, while six or seven animals would be sufficient to collect enough number of cells from the EOMs, EDL and tongue muscles, in case of the buccinator, due to its considerably low weight, a large number of animals, up to 32, would be necessary to get the same number of myogenic progenitors, failing to comply with the reduction principle first established by Russell and Burch in 1959 [52]. 

Trophic factors, like the neurotrophins, besides their important role during brain development, are also important regulators of muscle maintenance, function, and regeneration (reviewed by [27]). Accordingly, we studied by RT–qPCR the in vitro expression of BDNF, NGF and NT-3 on cultured myogenic progenitors derived from different muscles and compared it with the transcripts of EOMS-derived cells. Here, we describe for the first time, that rat EOM-derived cells show higher mRNA levels of BDNF, NGF and NT-3 as compared to their counterparts from the EDL. They also show higher levels of NGF and NT-3 as compared to cells derived from the tongue, and finally, they show higher levels of NGF as compared to cells derived from the buccinator muscle. 

Neurotrophins exert their actions through two classes of receptors, the tyrosine kinases family of receptors (Trk) and p75^NTR^, a receptor belonging to the tumor necrosis factor receptor family. There are three different types of Trk receptors: TrkA, TrkB and TrkC that show specificity for each type of neurotrophin; NGF binding specifically to TrkA, BDNF and NT-4 binding to TrkB, and NT-3 binding with high affinity to TrkC, but being also able to activate less efficiently the other two Trk receptors [22,53]. In order to understand the possible roles of neurotrophins in the process of myogenic regeneration, we investigated the expression of these receptors on the myogenic progenitors of different muscles. Thus, in accordance with what has been published before in C2C12 cells and human myocytes [31,54], the immunofluorescence analysis of TrkB or TrkC receptors in rat primary myoblast showed that neither of the cells compared here expressed these receptors in vitro. However, immunofluorescence analysis of rat myogenic cells from the EOMs, tongue, buccinators and EDL showed that the expression of the NGF high-affinity receptor TrkA is limited to the nucleus of these cells. This is not the first time that the predominant nuclear localization of this receptor has been described. Bonacchi et al. already demonstrated in 2008 [55] that in vivo hepatocytes and activated stellate cells from the liver expressed the NGF receptor preferentially in the nucleus of the cells, both in the presence and absence of injury, and they confirmed their finding in cultured hepatocytes. The expression of both, high- and low-affinity NGF receptors, TrkA and p75^NTR^ was already reported in L6 rat myoblasts, primary human myoblasts, and TE-671 rhabdomyosarcoma cells [56], as well as in developing adult rat myoblasts [57]. These studies show that NGF affects the proliferation, fusion into myotubes, and cell morphology of developing myoblasts. However, in human and mice myoblasts, as well as in murine cell lines, several studies show no expression of TrkA receptor and allude to p75^NTR^ regarding the beneficial effects NGF exerts over these cells [33,58,59,60]. These effects, although with several differences between the cells tested, include: modulation of myogenesis and differentiation, augmentation of fusion rates and dystrophin and MyHC isoforms expression, as well as muscle-protective responses to inflammation [33,54,58,60,61]. Here, the immunofluorescence analysis proved, for the first time, that EOM-derived myogenic cells show higher expression of p75^NTR^ as compared with cells derived from the EDL, tongue and buccinators muscles. Since the quantitative analysis of the neurotrophins transcripts also showed that these cells expressed higher levels of NGF mRNA, it would be interesting to test if their higher proliferative and fusion rates are related to an autocrine action through the NGF/p75^NTR^ pathway.

In summary, since the relevance of BDNF, NGF and NT-3 on different aspects of satellite cell function and muscle regeneration has been well documented [30,31,32,40,56], we suggest that higher expression of several neurotrophins and the p75^NTR^ receptor observed in EOM-derived progenitors, could be endowing these cells with their highly regenerative properties and thus could be protecting these craniofacial muscles against certain inflammatory and wasting diseases.

## Figures and Tables

**Table 1:** Comparison of different parameters measured on each type of muscle from which cells were isolated.

Parameter Measured	EOM	N	Buccinator	N	Tongue	N	EDL	N
Muscle weight (g)	0.20 ± 0.014●*‡	10	0.03 ± 0.004●†‡	4	0.49 ± 0.017*†	4	0.56 ± 0.045*†	3
N cells before plating (×10^5^)	2.88 ± 0.44*	10	0.63 ± 0.17	4	3.30 ± 0.6*	4	3.25 ± 0.19*	3
N cells on 1st passage (×10^5^)	2.92 ± 0.51*	7	0.68 ± 0.19	4	2.95 ± 0.65	3	3.17 ± 0.08*	3
N cells (×10^5^)/g muscle	1.45 ± 0.16*	10	2.21 ± 0.28	4	0.67 ± 0.12*†	4	0.59 ± 0.04*†	3
N rats to obtain 1 × 10^6^ cells	7	-	32	-	6	-	6	-

Values = means ± SEM on the left column, and *N* = number of independent cultures from which the data was obtained on the right columns of each type of muscle. Symbols indicate significant differences with tongue (dot, ●), extensor digitorum longus (EDL) (double cross, ‡), buccinator (asterisk, *) or extraocular muscles (EOMs) (cross, †). Two animals were used for each culture. One-way ANOVA, Holm–Sidak method for post-hoc comparisons, *P* < 0.05.
